# Spatiotemporal Characterizations of Spontaneously Beating Cardiomyocytes with Adaptive Reference Digital Image Correlation

**DOI:** 10.1038/s41598-019-54768-w

**Published:** 2019-12-05

**Authors:** Akankshya Shradhanjali, Brandon D. Riehl, Bin Duan, Ruiguo Yang, Jung Yul Lim

**Affiliations:** 1 Department of Mechanical and Materials Engineering, University of Nebraska-Lincoln, Lincoln, NE 68588 USA; 2 Mary & Dick Holland Regenerative Medicine Program, University of Nebraska Medical Center, Omaha, NE 68198 USA; 3 Division of Cardiology, Department of Internal Medicine, University of Nebraska Medical Center, Omaha, NE 68198 USA; 4 Nebraska Center for Integrated Biomolecular Communication, University of Nebraska-Lincoln, Lincoln, NE 68588 USA

**Keywords:** Regenerative medicine, Biomedical engineering

## Abstract

We developed an Adaptive Reference-Digital Image Correlation (AR-DIC) method that enables unbiased and accurate mechanics measurements of moving biological tissue samples. We applied the AR-DIC analysis to a spontaneously beating cardiomyocyte (CM) tissue, and could provide correct quantifications of tissue displacement and strain for the beating CMs utilizing physiologically-relevant, sarcomere displacement length-based contraction criteria. The data were further synthesized into novel spatiotemporal parameters of CM contraction to account for the CM beating homogeneity, synchronicity, and propagation as holistic measures of functional myocardial tissue development. Our AR-DIC analyses may thus provide advanced non-invasive characterization tools for assessing the development of spontaneously contracting CMs, suggesting an applicability in myocardial regenerative medicine.

## Introduction

Advanced tissue mechanical characterization and data visualization (Fig. S1) may lead to standardized measures of tissue mechanical functioning for *in vitro* tissues and for various *in vivo* diagnosis (e.g., ultrasound speckle tracking, magnetic resonance elastography, etc.). The characterization of spatiotemporal mechanical properties of cardiac tissue is especially important since the mechanical sensitivity of the heart is a recognized driver of cardiomyogenesis, homeostasis, and pathological hypertrophy^1,2^. For example, asynchrony of cardiac beating is clinically used for the assessment of cardiac health. Current myocardial tissue assessment methods with patch clamp, electrode array, microforce transducer, voltage-sensitive dye, etc., are invasive, require sophisticated instruments, or constrain the culture environment^3,4^. Since contraction quality is one of the most vital metrics to determine the functional health of cardiomyocyte (CM) tissue, alternatively, digital image correlation (DIC) may be used to assess the tissue via non-invasive imaging and monitoring. However, DIC involving correlation coefficient and median reference has only limited scope for biological samples^5–12^.

DIC is widely applicable to both hard and soft tissues at multiple length scales, and may be applied independently of mechanical tissue behavior (i.e., isotropicity, deformation range, etc.)^13^. The resolution and sensitivity of DIC depends on many factors including the imaging resolution, frame rate, and choice of correlation parameters. These factors must be tailored to capture the resolution at the tissue level. The non-contact nature of DIC excels for cell-based regenerative therapies since the tissues may be intended for transplantation^9^. However, the tissue must be visually accessible for imaging and subsurface tissue characterizations may be difficult or impossible for a simple imaging setup. DIC is also dependent on trackable features which may undergo non-affine deformations during contraction. Synthetic tracking molecules may be added to the tissue but, again, this is not ideal for an implantable tissue. Despite these limitations, DIC has been utilized as a powerful characterization tool for measuring the beating characteristics of individual sarcomeres, individual cells, and cardiac tissues. Quantifications using sarcomere banding, beating signal correlation with Ca^2+^, and dose-dependent drug responses are a sampling of the utility of DIC-based quantifications. See the review of Laurila *et al*.^14^ for a more detailed comparison of DIC to traditional CM quantifications.

**Figure 1:**
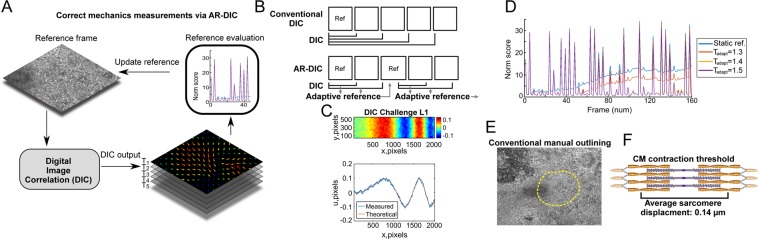
AR-DIC enables robust, unbiased, and accurate mechanics measurements of moving biological tissue samples such as spontaneously contacting CM tissue. (**A**) In AR-DIC, adaptive reference scheme continually adjusts reference frames enabling correct quantification of displacements occurring during the spontaneous contraction of cardiomyocytes. (**B**) Unlike conventional DIC depending on static reference frame, AR-DIC adaptively selects a new reference frame. (**C**) AR-DIC was validated using the 2D DIC Challenge 14-L1 of the Society for Experimental Mechanics (sem.org/dicchallenge/). AR-DIC (Measured) describes well the given test set (Theoretical). (**D**) Iterative refinement of the adaptive reference threshold (T_adapt_) reduces measurement error build-up, enabling robust DIC application to beating CMs. Finding T_adapt_ to enable long-term monitoring of contracting CMs required three iterations (T_adapt_ from 1.3 to 1.5). (**E**) Conventional manual outlining for beating area. (**F**) The average *in vivo* sarcomere displacement length of 0.14 µm was adopted in this study as a threshold over which displacement was considered as CM contraction^1^.

Here we developed Adaptive Reference-DIC (AR-DIC) to achieve robust, unbiased, and accurate kinematics and strain measurements of moving biological tissue samples, such as beating CMs, which lack stationary reference frames thus producing noise build-up during the measurement. We further defined novel spatiotemporal analyses, including heat maps that reveal the beating frequency and magnitude over time and position, and accumulative spatiotemporal maps that analyze interrelationships among moving tissue regions. In an application of AR-DIC (Figs. 1A and S1) to spontaneously beating CMs, engineered myocardial tissue development with measures of tissue maturation and homogeneity regarding spontaneous contraction were evaluated. We further demonstrate the utility for tracking tissue development using videos from Rajasingh *et al*.^6^ from three consecutive days. As a result, the “difficult-to-characterize” spontaneously beating CM tissue model could be analyzed in the localization, synchronization, and development of CM beating motion. The AR-DIC may further be utilized to examine the functional aspects of cardiac diseases via analyzing *in vitro* pathological hypertrophy model.

## Results

### AR-DIC enables robust non-invasive mechanics characterizations of beating CM tissue utilizing physiologically relevant contraction criteria

Inspired by optimization methods, our AR-DIC Matlab toolbox automatically selects a reference frame after major CM contraction cycle (Fig. 1B) in order to resolve the measurement error accrual in traditional DIC. The effectiveness of developed AR-DIC was tested by the DIC Challenge from the Society for Experimental Mechanics (sem.org/dicchallenge/) (Figs. 1C and S3). Through iterative refinement of adaptive reference threshold, AR-DIC applied to CM tissues could almost completely remove the measurement error build-up (Fig. 1D, T_adapt_ = 1.5), enabling robust application of DIC to assess the mechanics of CM contraction. Further, in contrast to manual outlining to define the contracting area (Fig. 1E), we adopted physiologically relevant criteria such that regions with displacement greater than the average individual CM sarcomere contraction length (0.14 µm) (Fig. 1F) were considered as “spontaneously beating”^1^. We adopted 0.14 µm as a guide based on a published sarcomere average displacement however other researchers may wish to select contraction based on the properties of their expected tissue displacement. This was achieved because AR-DIC was developed to have selection criteria adjustable and expandable. Of further utility, the adaptive reference scheme may be extended to other imaging modalities such as photoacoustic imaging, magnetic resonance imaging, magnetic resonance elastography, ultrasound, etc., that commonly lack standard reference frames.

**Figure 2:**
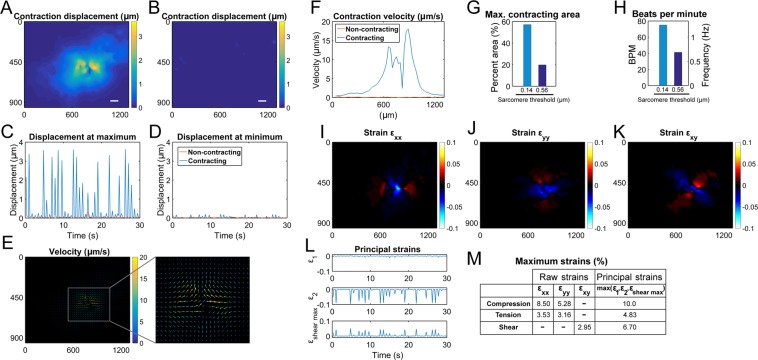
In AR-DIC of beating CMs, accurate displacement measurement and combined sarcomere length-based physiological contraction criteria provide multiple quantitative biomechanics data critical for assessing functional myocardial tissue development. Displacement at a time frame showing the maximum displacement for contracting (**A**) and non-contracting (**B**) samples, respectively. Scale bar is 100 µm. Displacement change with respect to time at the maximum (**C**) and minimum (**D**) displacement locations, respectively (figures include both contracting and non-contracting cases). (**E**) Velocity vector plot calculated from the displacement for the contracting sample. (**F**) A horizontal trace through the maximum velocity location shows contraction velocity change with distance from the contraction center. (**G**) Surface coverage percentage of beating area calculated at the maximum contraction area frame. Area with displacement >0.14 μm (sarcomere displacement length) was defined as contraction. Arbitrary threshold can also be set, e.g., displacement of four sarcomeres, 0.56 μm. (**H**) BPM calculated by FFT of displacement and verified with an automated peak counter is also based on the sarcomere length criteria. (**I**–**K**) Horizontal (ε_xx_), vertical (ε_yy_), and shear (ε_xy_) strains calculated from displacements for the contracting sample at the maximum displacement frame. Positive strains values are tensile and negative strains are compressive. (**L**) Principal strains over time at the maximum compressive strain location. (**M**) Maximum raw strains and maximum principal strains. Frame units in all relevant figures are in µm.

Measuring tissue displacement magnitude, beats per minute (BPM), and total contracting area may be a first step to assess contractile CM tissue health. The adaptive reference scheme enabled the correct measurements of tissue displacement during the CM beating (Figs. 2A–D, S4 and S5). The maximum displacement of 3.62 µm for the contracting sample (Figs. 2A,C) implies concerted effort of multiple (> two dozen) sarcomere units. Velocity field was then calculated from the displacement and time between frames (Fig. 2E). A horizontal trace through the maximum velocity position reveals that velocity changes from the contraction center (Fig. 2F). The sarcomere displacement length criteria (0.14 µm) enabled physiology-based quantifications of contracting area (Fig. 2G) and BPM (Fig. 2H) opposed to conventional manual drawing and counting. The beating area, assessed by the area showing displacement more than one sarcomere displacement (0.14 μm), had a maximum field of view coverage of 57.2% (or 6.99 × 10^5^ µm^2^), indicating an ongoing engineered myocardial tissue development as the tissue was assessed on day 10 of CM differentiation. Our method can also set other arbitrary contraction thresholds to define CM beating, e.g., four sarcomere displacement length (0.56 μm). This allows the method to be adapted to each study based on the anticipated displacements and available imaging resolution. BPM, calculated by the fast Fourier transform (FFT) of the displacement signal and verified with an automated peak counter, was 75.0 for the beating areas having displacement more than 0.14 μm; which is comparable to BPMs in previous studies including ours^2,14,15^.

AR-DIC quantifications next enable the calculation of the tissue strain. Strain is known to play a critical role in the mechanical feedback loop assisting in cardiac development and homeostasis^16^. Compressive strains were observed at the contraction center and tensile strains were present on either side of the compressive region (Figs. 2I,J and S6). The shear strain had regions of opposing shear rotating away from the contraction center (Fig. 2K), which resembles the twisting of *in vivo* cardiac tissue during contraction^16^. Principal strains at the maximum compression location (Fig. 2L) and values of maximum principal strains (Fig. 2M) may provide magnitude information on *in vivo* myocardial tissue strains during the heart beating. Plotting transformed principal strains indicates a central region with compressive strains (Fig. S7). Having compressive strains implies a net decrease in the local tissue area, which could potentially function as a contraction origin (related strain data in Figs. S8 and S9). For functional myocardial tissue engineering, mechanical loading at these identified strains (up to about 10%) and frequencies (up to about 1.25 Hz corresponding to 75 BPM as noted above) may therefore be attempted for physiological relevancy consideration (as we adopted^15^).

### Spatiotemporal data exploration and visualization assess myocardial tissue contraction homogeneity, interdependency, synchronicity, and propagation

**Figure 3:**
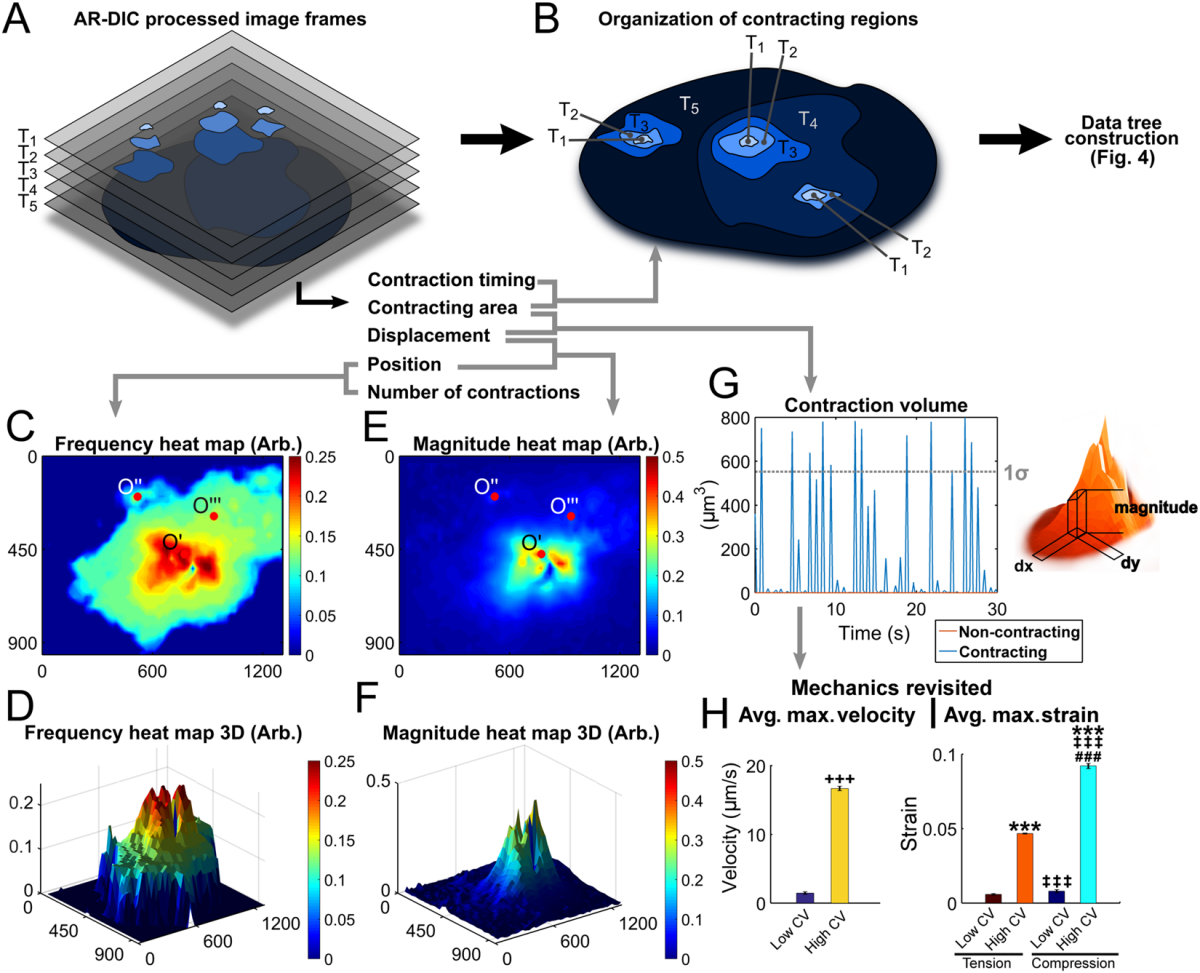
Holistic measures of functional myocardial tissue development assessing the homogeneity of CM tissue contraction can be achieved by AR-DIC with newly developed spatiotemporal parameters. Contracting regions arranged in spatial layers over time (**A**) and overlaid as a single layer (**B**) (schematic illustrations). (**C**–**F**) Contraction frequency and magnitude heat maps are newly defined to display spatial beating parameters accumulated over time. Contraction origins identified in ASC and ASTC maps (see Fig. 4) are marked as O′, O″, and O″′. (**G**) Contraction volume (CV) for the entire viewing field, accounting for both beating area and displacement magnitude, is plotted over time. A dotted line denotes 1σ below the maximum CV. (**H**,**I**) Average maximum velocity and strain plotted for high and low CV regions. High CV region was defined as to be within 1σ below the maximum CV, below which was considered as low CV. Mean ± standard error of measurement. ^+++^p < 0.001 compared with low CV; ***p < 0.001 with low CV tension; ^‡‡‡^p < 0.01 with high CV tension; ^###^p < 0.001 with low CV compression. Frame units in all relevant figures are in µm.

Next, the homogeneity, propagation, and synchronicity of CM contraction which have significant clinical implications to evaluate the functional health of myocardial tissues^17,18^ could be evaluated. AR-DIC processed images were arranged in spatial layers over time (Fig. 3A as schematic), from which spatial overlap of beating regions was analyzed (Fig. 3B). As a new spatial assessment of CM beating, heat maps for beating frequency and displacement magnitude were constructed (Fig. 3C–F). Interestingly, centers of beating frequency and magnitude are not always coincident (or, the most frequently beating region is not necessarily that having the largest displacement). The contraction volume (CV) (Fig. 3G) was quantified to provide a more holistic measure of CM beating by combining beating area and displacement. When mechanics data are revisited with CV, regions with high CV (within 1σ below maximum CV) displayed significantly greater velocity (Fig. 3H) and larger strains (Fig. 3I) relative to regions with low CV. These quantifications provide a metric of contraction development that may be tracked for an individual tissue region over time or used for comparisons among tissues.

**Figure 4:**
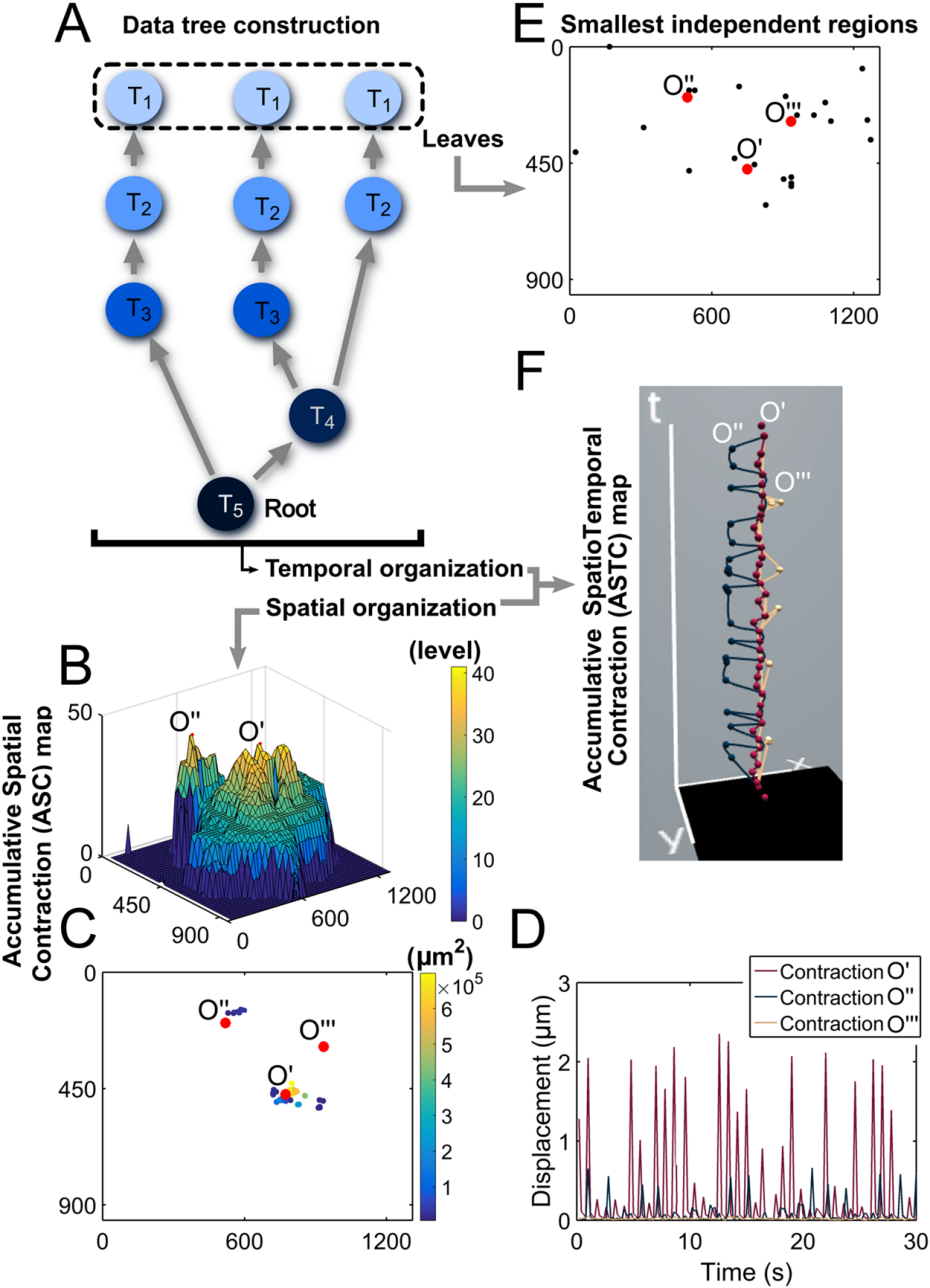
Tree data analysis-based novel spatiotemporal data exploration and visualization assess myocardial tissue contraction in interdependency, synchronicity, and propagation. (**A**) A tree data structure is defined to determine contraction origins and the interdependency between contracting regions. The contraction origin is considered as the leaf (at time T_1_) belonging to one big contracting area (at time T_5_) which is the root of the data tree. (**B**) The ASC map plotting the depth of the tree data distinguishes contracting regions based on their functional beating activity. More yellowish color regions having more depth in data tree can represent the primary beating areas (or contraction origins), while green then blue areas are neighboring regions contracting due to the contraction waves originating from the primary regions. The highest (O′) and second highest (O″) peaks are marked in the 3D ASC map (the third highest O″′ is not seen due to the location). (**C**) Three major contracting origins (O′, O″, O″′) are shown as well as centroids of other contracting regions for those larger than 10 DIC vector units or 4840 µm^2^. Centroids are color-coded to show the contracting area (µm^2^). Centroids around O″′ are not seen due to contracting area size or overlapping with O″′. (**D**) Displacements at O′, O″, and O″′ are plotted over time. (**E**) The data tree analysis also identified small independently beating regions (black dots), indicating there are also contractions less dependent of the primary contractions at O′, O″, and O″′. (**F**) The ASTC map representing the contracting data tree through space and time axes visualizes the interaction of the contraction origins. This map thus illustrates the synchronicity and propagation of the beating among the contraction origins. The primary contracting origin (O′) is the most active, and the contraction timing between O′ and the secondary (O″) and tertiary (O″′) origins are evident along the vertical time axis. Frame units in all relevant figures are in µm.

We further developed novel tools to explore spatial and temporal relationships among contracting regions. Data tree structure was adapted to determine the contraction origin and interdependency among beating regions (Fig. 4A). Each contracting region was represented as a node, and the parental lineage of the nodes was assigned until all nodes were included in the tree. In this tree analysis, leaf nodes (T_1_) could be identified as contraction origins, which belong to one big contracting area (T_5_) termed as the root. Accumulative Spatial Contraction (ASC) map (Fig. 4B) was defined to identify contraction origins and spatially illustrate the interdependency of contracting regions. The *z*-axis of the 3D ASC map was encoded to represent a chain of influence, corresponding to the depth of the data tree nodes (Fig. 4A). Thus, elevated peaks in the ASC map can be identified as the origins of contraction. Observing the overlapping bases of such peaks, the contracting regions are not entirely independent as the overlapping area can be affected from multiple peaks. The ASC map reveals three distinct contraction origins (Fig. 4B), i.e., O′ having the deepest tree architecture or highest peak in the 3D ASC map being the strongest contracting origin; O″ the secondary; O″′ the tertiary (though not seen in 3D view due to its location), and around these origins their dependent beating regions are shown (Fig. 4C). The displacements of these three origins can be seen in Fig. 4D. There were also found small independently beating regions in the tree analysis identified as the remainder of the leaves in the data tree (Fig. 4E). Accumulative SpatioTemporal Contraction (ASTC) map (Fig. 4F) extends this tree data organization and presentation to an additional dimension, time. In ASTC map, each contracting origin is plotted as a node in space and time dimensions. The mechanical wave propagation between origins can be seen in the horizontal plane and the contraction timing and synchronization can be observed along the vertical axis, from which spatiotemporal relationships between contraction origins are observed (see Fig. S10 for beating propagation and synchronization among all identified contracting areas). Additionally, new visualization methods were developed to assist the viewing of large datasets intuitively, such as displacement seen as topography (Fig. S11).

## Discussion

DIC with adaptive reference selection criteria proved to be effective as a non-invasive tool for ongoing evaluation of functional tissue mechanical properties. Further, the AR-DIC method provided high quality data so that advanced spatiotemporal characterizations were possible. Previous imaging-based approaches to CM beating characterization have used block matching, texture movement, correlation coefficient-based methods, and the median reference frame method^4–11,14^. Ahola *et al*.^5^ devised an image correlation method for individual CMs segmented by the user to quantify the beating signal. Obtaining similar beating measurements for CM monolayers, Huebsch *et al*.^10^ applied an optic flow block matching algorithm to automatically assess beating rate to remove user bias from selecting regions of interest. Also using optic flow, Kamgoué *et al*.^8^ assessed the strain in individual CM sarcomeres. These previous approaches have either relied on a static reference or compared the current image frame to the subsequent frame resulting in only relative measurements. These tissue characterization methods can have a common limitation due to the lack of reference frames and thus may not be suitable for ongoing *in-situ* monitoring of functional tissue development, or have limited scope such that a complex interplay of spatial and temporal contraction patterns could not be elucidated. Our work departs from previous processing schemes by establishing a baseline from which absolute displacement measurements can be made. Our method is pacing-agnostic so that it can be used for spontaneously contracting CMs. If used with pacing, our method eliminates the need to obtain the reference frame from pacing data. In a study measuring image pixel intensity change (but not tissue displacement), Sala *et al*.^19^ also noted the benefit of an automated baseline selection by finding the image frames corresponding to a diastolic period. We note that the MuscleMotion program by Sala *et al*.^19^ may be a good option for researchers who need to quantify the contraction magnitude vs. time while not requiring displacement, strain, or other spatiotemporal quantifications as our method could provide. Investigation of further criteria and reference selection logic will greatly benefit the DIC application to different cases and improve the robustness of DIC and its computation efficiency. The current evaluations have resulted in a single-factor optimization function, and more sophisticated methods may rely on multiple factors to achieve optimal results.

Tissue development and homeostasis are dependent on mechanical factors including the distribution of stresses and strains, loading modality (e.g. stretch, fluid shear, compression), and tissue viscoelastic properties. Our computational and visualization methods characterize mechanical tissue health and functionality which may assess the functional development, heterogeneity, and maturation of tissue, and can be applied to analyze dysfunction in mechanically-altered diseases. Our velocity (Fig. 2E) and strain data (Fig. 2I–K) indicate that the center of the contraction region (O′, Fig. 4B) is responsible for driving the contraction force and mechanical impulses in and around the mechanically active tissues. As the site of contraction initiation, this region may also serve in a similar manner as the sinoatrial node *in vivo*. Basic parameters such as BPM and contracting area were found to be similar to our previously published results^15^. Interestingly, from our results the strains and beating frequency of the spontaneously contracting CMs are within the range to enhance CM induction through mechanical means. The CMs likely experience a mechanical feedback loop assisting in further differentiation and maintenance of homeostasis.

Spatiotemporal distribution of CM contraction defined in our study could be established as an important determinant of normal functioning of CM tissues for high throughput analysis and evaluating their mechanobiological characteristics. From frequency and magnitude heat maps, high activity regions are more obvious than in simple displacement plots, and secondary contracting areas can be identified, indicating different regions are contracting at different degrees of frequency and at varying magnitudes. The homogeneity and synchronicity of CM beating may be improved with increased culture duration, which can be detected using the proposed non-invasive measurements of the ASTC map (Fig. 4F), contraction volume (Fig. 3G) or frequency and magnitude heat maps (Fig. 3C–F). This information can be used to assess if the beating CMs are fully developed in functional contractility. Contraction roots were also identified and the interaction was visualized in the ASC and ASTC maps. From these maps it is clear that the roots are not entirely independent. In a mature tissue these contraction roots synchronize and form a homogenous contracting region. This results in a contracting tissue root having only one associated contraction leaf in the data tree. This is because contraction would be initiated in one centralized location. The presence of multiple leaves is a sign of discord in spatial contraction homogeneity. These may be due to the ongoing development of contractility in that region of the tissue and the contraction may not be relatively synchronized with other contraction roots thus leading to the many leaves. With this information, the tissue development can be quantified and tracked in terms of contraction root progression, synchronization, and integration in the data tree. Further, applying these methods to *in vitro* disease models will provide additional insight to quantify mechanical dysfunction. Together the novel DIC methods and tissue characterizations provide researchers and clinicians non-invasive tools for mechanobiology assessment.

Cardiac tissue organization and structure has a direct effect on tissue health and function^16^. The organization of contractile units, extracellular matrix, and electrically coupled gap junctions contribute to pathological hypertrophy and the presence of heterogeneities are associated with electrical disorders^20^. Using our functional mapping capabilities, localized tissue sampling and stimulation of the contraction origins, primary contracting regions, and secondary contracting regions will then be possible. Our methods can integrate with and complement current micro electrode array methods for identifying pacemaker regions and electrical heterogeneity. These characterizations will enable better models to be constructed with sampling of parameters from the developing tissue whenever necessary. This is especially necessary for cardiovascular tissues which are inhomogeneous and may require sampling at multiple scale levels to achieve a sufficiently detailed model^21^. This will better inform developmental models and enable testing of hypotheses at distinct developmental tissue stages. Further, data predictions from modeling can be used as feedback to stimulate changes in the developing tissue. For example, location, timing, and synchronization of contraction origins may be influenced to either guide tissue development or simulate disease states.

In summary, our non-invasive holistic CM characterizations can complement current myocardial assessment tools, providing additional insight, visualization, and parameters to better inform myocardial tissue engineering and disease studies. Characterization of spatiotemporal CM beating based on accurate mechanics measurement, sarcomere-based physiological contraction criteria, and newly defined tree data structure may be established as an important determinant of healthy functioning of myocardial tissues for non-invasive and high-throughput analysis. AR-DIC does require basic knowledge of DIC principles for successful application. Users may also elect to be creative on applying AR-DIC to only specific regions of interest such that different regions may have different reference frames. Our methods are readily available in a Matlab toolbox and may be adapted to other biological imaging modalities (as noted above). For example, AR-DIC was further validated using other published CM differentiation model (Figs. S12 and S13). This demonstrates the utility of AR-DIC for tracking tissue development over time with quantitative parameters. Data non-invasively obtained through AR-DIC may thus be utilized to optimize CM developmental protocols for myocardial tissue engineering and further assist myocardial pathology studies.

## Materials and Methods

### Spontaneously beating CMs

P19 murine embryonal stem cell-like carcinoma cells from ATCC (CRL-1825) were used to induce contracting CMs, as we recently reported^15^. Protocols used for this induction are described in the Supplementary Information section. All methods were carried out in accordance with relevant guidelines and regulations of our university’s Institutional Biosafety Committee (IBC).

### Video imaging and data acquisition

Time lapse image stacks of spontaneously beating CMs and non-beating control were obtained. A Leica DMI 4000B microscope with 10X objective was used to record Video images at 5 frames per second (FPS) with a Photometrics CoolSnap EZ camera having 1392 × 1040 pixels. A frame rate of 5 FPS is sufficient to assess the basic BPM quantification up to about 150 BPM, as calculated based on the Nyquist sampling theorem^22^. In our study with the tissue having 75 BPM, 5 FPS provides enough time resolution to extract the beating signal. The frame rate should be chosen such that the sampling frequency is twice that of the expected feature frequency. Laurila *et al*.^14^ noted that for applications such as detecting drug-induced changes in the contraction-relaxation phase, higher frame rates of 125 FPS may be necessary. Practically, the sampling rate must take into account the processing time. A frame rate of 5 FPS was sufficient to describe the overall contraction behavior of our spontaneously contracting CMs. Videos S1 and S2 are provided for the contracting and non-contracting samples, respectively (see the Supplementary Information for links).

### Adaptive Reference-Digital Image Correlation (AR-DIC)

A controlled static reference frame for spontaneously beating CMs normally does not exist. The need to analyze time varying signals also prohibits the use of a static reference frame because of the noise build-up due to minor frame shifts after each contraction cycle. To resolve these, we developed a method to automatically choose a reference frame after each contraction cycle. While the application in this study is for beating CMs, AR-DIC scheme can be widely applicable to various biological samples that lack clear stationary reference frames.

We wrote Matlab scripts packaged into a Matlab toolbox to control FIJI/ImageJ^23^ software and to adaptively select a reference frame from the time lapse image stacks. Briefly, an initial reference frame without visible contraction is selected as a primary reference frame for DIC. As a starting point, the initial reference frame which lacks visible contraction can be selected manually. This initial reference and the value of T_adapt_ can later be refined iteratively based on the output from DIC. For Video S1, frame 1 was chosen in this study. Matlab then sends this reference and the current analysis frame to the ImageJ Iterative Particle Image Velocimetry (PIV) (Advanced) plugin^24^. The output displacement field is analyzed to determine if the current frame is a suitable reference frame or not for future contraction cycles. To determine this, the Frobenius norm is calculated from the displacement matrix. The reference frame selection occurs if the Frobenius norm from the current DIC iteration is below the adaptive reference threshold (T_adapt_). The value of T_adapt_ represents the noise floor or latent displacement of the reference frames. The initial starting value of T_adapt_ is obtained by estimating the Frobenius norm score of two potential reference frames. To estimate T_adapt_ for Videos S1, we selected the initial reference frame and a second frame without visible displacement (e.g., frame 1 and 3, respectively). The Frobenius norm of displacement between frame 1 and 3 was 1.25, which yielded our starting T_adapt_ estimate of 1.3 when rounded. Then, T_adapt_ is finalized via iterative refinement. In this study, three iterations were sufficient (T_adapt_ = 1.3, 1.4, 1.5) to achieve the baseline norm score to remain constant (Fig. 1D). Such an iterative refinement of the adaptive reference threshold could remove measurement error build-up, enabling robust DIC application to contracting CMs. If needed, other selection mechanisms besides the Frobenius norm, e.g., minimization of an objective function, can also be easily implemented. We designed Matlab data processing and visualization code in a modular object oriented paradigm, so that it is extensible and adaptable for other imaging devices and tools, even smartphones. See the attached AR-DIC User Manual for full code documentation, instruction for installation, and demos.

The iterative PIV (Advanced) plugin in ImageJ makes use of multiple passes through the image frames. The algorithm uses progressively more detailed search settings depending on the window size and vector spacing to capture the essential features during each iteration (Table S1). The results of the previous iteration feed forward to seed the next search at higher resolution. The cross-correlation method is used to ensure agreement between successive passes. This procedure results in PIV output with a higher resolution and is able to provide subpixel results. While PIV has been typically applied to quantify the metrics of fluid flows in successive image frames^25^, our Matlab control of the reference frame could provide a versatile method for many moving biological samples. In this study, such assessments were conducted with a vector spacing of 24 pixels, which captured sufficient details of CM beating. The plugin was also tested with a vector spacing of 2 pixels (Fig. S13). However, this did not significantly increase the useful data output while only increasing the calculation time.

To run FIJI within Matlab, we used the MIJ portion of the MIJI project^23^. This enabled Matlab to send commands to FIJI. The setup was tested on Windows7, Windows10, and MacOS Sierra. On Windows, this required enabling of the Java 8 update site in FIJI. Matlab R2017a and later releases natively use Java 8. A system variable can be set to use Java 8 with earlier versions of Matlab. The FIJI scripts folder was then added to the Matlab path. The MIJ.jar and IJ-x.jar were added to the Matlab Java and the MIJ.jar path added to the Matlab Java path. Finally, “iterative PIV (Advanced)” plugin could be installed to the FIJI version associated with Matlab.

### Validation of AR-DIC

Developed displacement measurement methods based on AR-DIC were validated with the “2D-DIC Challenge Sample 14” from the Society for Experimental Mechanics (sem.org/dicchallenge/). The Sample 14 is a synthetic dataset created with FFT transform to cause pixel shifts of known displacements. The dataset consists of a reference and a deformed image with a known sinusoidal deformation at increasing strain gradient. The Sample 14 also includes considerable noise to test the robustness of the DIC. The parameters used in our PIV plugin calculations for the DIC Challenge are shown in Table S2.

### CM beating defined by sarcomere contraction length

Our AR-DIC was designed to set any arbitrary threshold value to define the CM contraction. To set physiologically relevant criteria, we evaluated such that the region showing displacement greater than 0.14 µm, the CM sarcomere displacement length^1^, is participating in spontaneous beating. Using this condition, beating area could be quantified by not depending on manual drawing. BPM could be also calculated via FFT of the displacement signal for the areas with displacement greater than 0.14 µm and verified with an automated peak counter, not via manual counting from the video.

### Heat maps for CM beating frequency and displacement magnitude

As new spatiotemporal measures of CM beating, frequency and magnitude heat maps were created. The frequency heat map was produced by setting a binary mask for each frame with thresholding the displacement matrices with a criterion of displacement greater than the physiological sarcomere contraction length, 0.14 µm. The heat map was constructed by adding together such binary masks and normalizing with the number of video frames, thus it is defined as normalized number of times each region contracts throughout the video. In the color-coded heat map, the high activity areas are more obvious and secondary contracting areas could be identified. In a similar fashion, the heat map for the contraction magnitude was obtained by adding the matrices of displacement throughout the video and normalizing with the number of frames.

### Contraction volume (CV)

Contraction volume takes into account both the area and magnitude of contraction. The sum of the displacement magnitudes was multiplied with the contracting area at that magnitude, which allows a single number to display relative CM contractile properties. The CV calculation was normalized by the DIC vector block size (532.6 µm^2^). “High CV” was defined as the frames within one standard deviation (1σ) below the maximum CV value (dotted line in Fig. 3G), while “Low CV” as the frames with CV less than this threshold. For the high and low CV areas, the maximum velocity and strain displayed significant differences.

### Tree data construction

In addition to mechanics measurements, temporal and spatial dynamics of contracting CMs were computed. A data tree analysis was implemented to find interrelationships between the contracting regions in both time and space axes. In general, tree data structures are used for their convenience in data accessibility, sorting algorithms, and maintaining connections between data points as in network analysis. Tree data structures have proved to be useful for organizing biological data such as in mapping the intrathoracic airway^26^. To organize the CM beating data into the data tree, the contracting regions as assessed by the sarcomere displacement length criteria were represented as nodes in the tree data structure. Node parental lineage was assigned beginning with the smallest regions and continuing until all contracting nodes were included in the tree. A node is considered a parent node if the region includes the centroid of the child node. From this procedure, whether each region is contracting independently or one region influences multiple contracting regions could be evaluated. A node without any children is called a leaf, and such leaf nodes can be considered as potential contracting origins (or small independent beating regions if they are not connected with other nodes).

### Accumulative spatial contraction (ASC) map

The ASC map is a visualization of the spatial information in the tree data structure. The tree is traversed starting at the root and traveling to each subsequent node. The *z* axis of the 3D ASC map encodes the depth level in the data tree, which is interpreted as the level of influence the region may have over the other regions. In such analysis, the highest mountainous positions in the 3D ASC map (or nodes with the deepest tree hierarchy) tend to play a role as the origins of contraction for the CM beating.

### Accumulative spatiotemporal contraction (ASTC) map

The ASTC map visualizes both the spatial and temporal information among potential contraction origins. The entire data tree/path of contraction may be visualized or individual paths from selected nodes or leaves can be illustrated using our tools. Inspecting the ASTC map in 3D view can reveal the localization of the contracting nodes and also the timing between contraction events. Thus, beating propagation or synchronicity among selected beating areas can be more intuitively pictured. Independent nodes would also be visible as the nodes disconnected from the main contracting paths. This visualization was built in the Processing open source programming language (version 3.3.4)^27^. Obtained tree data structure was exported from Matlab in a CSV format to be imported into Processing 3.3.4.

## Supplementary information

Supp. Info.

## Data Availability

All data, code, and materials used in the analysis including the user manual are available in the Supplementary Information and at: https://github.com/TheLimLab/AR-DIC.
