# Supplementary material for: Spatiotemporal Characterizations of Spontaneously Beating Cardiomyocytes with Adaptive Reference Digital Image Correlation
Source: Sci Rep. 2019 Dec 5;9:18382. doi: 10.1038/s41598-019-54768-w (PMC6895104; doi:10.1038/s41598-019-54768-w)
Supplement: Supplementary file 1 — Supp. Info. [file 41598_2019_54768_MOESM1_ESM.pdf]

Supplementary Materials for

Spatiotemporal Characterizations of Spontaneously Beating Cardiomyocytes with Adaptive Reference Digital Image Correlation

Akankshya Shradhanjali, Brandon D. Riehl, Bin Duan, Ruiguo Yang & Jung Yul Lim

Correspondence to: J.Y.L. (jlim4@unl.edu)

This PDF file includes:

Supplementary Text
Figs. S1 to S15
Tables S1 to S2
Links for Videos S1 to S2

Other Supplementary Materials for this manuscript include the following:

Videos S1 to S2
Supplementary Code and Demos
User Manual for AR-DIC Code

Supplementary Text

Table S1. Iterative PIV (Advanced) settings (correlation = 0.8).

Pass number	Interrogation window (pixels)	Search window (pixels)	Vector spacing (pixels)
1	128	256	64
2	64	128	32
3	48	96	24

Table S2. Parameters for ImageJ PIV plugin.

Pass number	Interrogation window (pixels)	Search window (pixels)	Vector spacing (pixels)
1	128	256	8
2	64	128	4
3	48	128	2

Induction of spontaneously beating CMs from murine multipotent cells

As we recently reported[15], P19 murine embryonal stem cell-like carcinoma cells (ATCC, CRL-1825) were used to produce beating cardiomyocytes (CMs). Briefly, cells were cultured for 4 days in bacterial grade petri dishes at 1×10^6 cells/ml to form embryoid bodies (EBs) using growth media consisting of Dulbecco's modified Eagle's medium with 10% fetal bovine serum, 1% penicillin-streptomycin, and 1% dimethyl sulfoxide. Formed EBs were transferred to type-I collagen-coated silicone membrane (Bioflex 6-well plates, Flexcell) at around 200 EBs per well and allowed to adhere for 24 h. Then, cyclic stretch loading (equiaxial stretching at 12% strain and 1.25 Hz for 24 h) was applied to the EBs seeded on the stretchable membrane using the Flexcell FX-5000 Tension system which controls a vacuum pressure to deform the membrane. After 24 h of stretch loading inside the incubator, P19-derived EBs were maintained with the growth media changed every two days. As we reported[15], these EBs exposed to stretch loading showed spontaneous contraction from about day 10. For Adaptive Reference-Digital Image Correlation (AR-DIC)-based spatiotemporal analyses of CM beating, these EBs on day 10 were used as a "spontaneously contracting" sample. As a "non-contracting" control group, EBs seeded and maintained the same on the Bioflex plates for 10 days but not exposed to stretch loading were used.

Kinematics and strain assessments of CM contraction

An interpreter was scripted to read output DIC files from the iterative PIV (Advanced) plugin. The program was organized into objects with each containing data structure, calculation methods, and visualization functions. Given a folder of the output DIC files, each file was read in order and the relevant displacement information was extracted and placed in a data structure. All data were scaled for pixel/µm conversion, DIC vector spacing, and time between frames. Using these, the displacements and velocities were calculated, and strains were computed from the displacement matrices. In strain assessment, the Green-Lagrangian strain tensor was calculated from which the strain at any arbitrary position could be determined.

Furthermore, to trace the change of strains over time, the principal strains at the maximum compressive strain location could be plotted with respect to time (**Fig. 2L**). Such assessment of maximum compressive strain location may provide useful mechanics knowledge about the contraction origin of CM beating. The capability of monitoring strains throughout the contraction cycles, and potentially during tissue development, will provide a useful tool for gauging tissue maturity in the process of myocardial tissue engineering. Considering the magnitude of strains is critically important for myocardial functioning both during development and later homeostasis[16], these calculations can provide vital information on the strain environments in terms of maximum tensile and compressive magnitudes and shear motion due to shape distortion related to CM contraction.

Green-Lagrangian strain calculations

We here present the Green-Lagrangian strain calculations in tensor and matrix format with 'x' corresponding to the horizontal direction (from the imaged view) and 'y' to the vertical direction. The displacement vector field, S, with horizontal and vertical displacement components u and v, respectively, describes the displacement for every sample point:

$$S(x, y) = \begin{Bmatrix} u \\ v \end{Bmatrix} \tag{1}$$

The deformation gradient tensor, *F*, is then calculated from the displacement field (*S*):

$$F = \nabla S = \begin{bmatrix} \frac{\partial u}{\partial x} & \frac{\partial u}{\partial y} \\ \frac{\partial v}{\partial x} & \frac{\partial v}{\partial y} \end{bmatrix} \tag{2}$$

where ∇ is the gradient operator and $\partial u/\partial x$, $\partial u/\partial y$, $\partial v/\partial x$, and $\partial v/\partial y$ are the matrices containing the respective gradients. The Green-Lagrangian strain tensor, *E*, calculated from the deformation gradient (*F*), is defined by

$$E = \frac{1}{2}(F^T \cdot F - I) \tag{3}$$

where *T* is the transpose operator and *I* is the identity tensor. For the two-dimensional case, the form of the strain tensor is

$$E = \begin{bmatrix} Exx & Exy \\ Exy & Eyy \end{bmatrix} \tag{4}$$

where the tensor components *Exx*, *Eyy*, and *Exy* and are the horizontal, vertical, and shear components of the Green-Lagrangian strain tensor, respectively. Since the tensor is symmetric, the notation *Exy* is used for both shear components. Expanding Equation (3), the tensor components could be obtained from the displacement gradient tensor components as below:

$$Exx = \frac{\partial u}{\partial x} + \frac{1}{2}(\left(\frac{\partial u}{\partial x}\right)^2 + \left(\frac{\partial v}{\partial x}\right)^2) \tag{5}$$

$$Eyy = \frac{\partial v}{\partial y} + \frac{1}{2}(\left(\frac{\partial u}{\partial y}\right)^2 + \left(\frac{\partial v}{\partial y}\right)^2) \tag{6}$$

$$Exy = \frac{1}{2}(\frac{\partial u}{\partial y} + \frac{\partial v}{\partial x}) + \frac{1}{2}(\frac{\partial u}{\partial x}.\frac{\partial u}{\partial y} + \frac{\partial v}{\partial x}.\frac{\partial v}{\partial y}) \tag{7}$$

The tensor components *Exx*, *Eyy*, and *Exy* are two dimensional matrices containing individual strain components ε_{xx}, ε_{yy}, and ε_{xy} as shown in Equations (8-10). For an $n \times m$ sample, the tensor components have the form:

$$Exx = \begin{bmatrix} \varepsilon_{xx_{1,1}} & \varepsilon_{xx_{1,2}} & \cdots & \varepsilon_{xx_{1,m}} \\ \varepsilon_{xx_{2,1}} & \cdots & \cdots & \varepsilon_{xx_{2,m}} \\ \cdots & \cdots & \cdots & \cdots \\ \varepsilon_{xx_{n,1}} & \cdots & \cdots & \varepsilon_{xx_{n,m}} \end{bmatrix} \tag{8}$$

$$Exy = \begin{bmatrix} \varepsilon_{xy_{1,1}} & \varepsilon_{xy_{1,2}} & \cdots & \varepsilon_{xy_{1,m}} \\ \varepsilon_{xy_{2,1}} & \cdots & \cdots & \varepsilon_{xy_{2,m}} \\ \cdots & \cdots & \cdots & \cdots \\ \varepsilon_{xy_{n,1}} & \cdots & \cdots & \varepsilon_{xy_{n,m}} \end{bmatrix} \tag{9}$$

$$Eyy = \begin{bmatrix} \varepsilon_{yy_{1,1}} & \varepsilon_{yy_{1,2}} & \cdots & \varepsilon_{yy_{1,m}} \\ \varepsilon_{yy_{2,1}} & \cdots & \cdots & \varepsilon_{yy_{2,m}} \\ \cdots & \cdots & \cdots & \cdots \\ \varepsilon_{yy_{n,1}} & \cdots & \cdots & \varepsilon_{yy_{n,m}} \end{bmatrix} \tag{10}$$

with *n* and *m* indices corresponding to the location in the measurement field and the ellipses implying the continuation of the indices. Note, the strain tensor components are distinguished with a capital *E* and the actual strains are notated with ε.

The strains (ε_{xx}, ε_{xy}, ε_{yy}) can be now calculated for every location of the sample in the current coordinate system. As the determination of the *x* and *y* axes of the coordinate system is arbitrary, it would be more instructive to find a coordinate transform to obtain the maximum tensile, compressive, and shear strain in the sample. These maximum strains, known as the principal strains, are invariants and the concept applies in both tensor (*E*) and scalar (ε) forms. The maximum strain is notated, ε_1 and the corresponding minimum strain is ε_2. To obtain ε_1 and ε_2, the mean strain, ε_m, is obtained in the first place:

$$\varepsilon_m = \frac{(\varepsilon_{xx}+\varepsilon_{yy})}{2} \tag{11}$$

The mean strain is the normal strain, i.e., the strain acting along the coordinate axis and is responsible for the change in sample area. Next, the maximum shear strain is calculated as

$$\varepsilon_{shear\,max} = \sqrt{\left(\frac{(\varepsilon_{xx}-\varepsilon_{yy})}{2}\right)^2 + \left(2\varepsilon_{xy}\right)^2} \tag{12}$$

which is the deviatoric strain, i.e., strain due to shape distortion. From these results the maximum strain, ε_1, is calculated as

$$\varepsilon_1 = \varepsilon_m + \varepsilon_{shear\,max} \tag{13}$$

And the minimum strain, ε_2 as

$$\varepsilon_2 = \varepsilon_m - \varepsilon_{shear\,max} \tag{14}$$

Finally, the principal angle, θ_p, which defines the coordinate rotation required to obtain this transform is found by solving

$$\tan 2\theta_p = \frac{2\varepsilon_{xy}}{\varepsilon_{xx}-\varepsilon_{yy}} \tag{15}$$

Using Equations (1-15), consequently, we could determine the strains (ε_{xx}, ε_{xy}, ε_{yy}) at any location in the sample, the maximum and minimum principal strains (ε_1, ε_2) with corresponding principal angle coordinate rotation (θ_p) required, and the maximum shear strain ($\varepsilon_{\text{shear max}}$). By convention, we consider positive normal strains as tensile and negative strains as compressive.

<u>Synthetic topography for CM beating visualization</u>

To intuitively visualize CM contraction, an export function was written to translate the displacement matrices into a format readable by the Tangram software (Mapzen.com/tangram). Map tiles were generated by encoding the surface normal of the displacement data to the RGB channels of a PNG image tile. The PNG alpha channel was scaled from sea level to 1200 m (Mt. Diablo, CA, USA). Map shading was similar to the Imhof scheme in the Tangram terrain demos

(https://github.com/tangrams/terrain-demos). The Tangram software was run in a Chrome web browser using the Python scripting to run a local HTTP server. With this setup all frames can be explored as a landscape with significant displacements of CM contraction appearing as mountainous features.

Comparison with previous publication

To further verify our AR-DIC method, from the publication by Rajasingh et al.[6] the file “S2 Movie. Day 8 high frame rate video microscopy image of iPSC-CMs used for measuring contractility” was downloaded and tested. The AVI file was converted to an uncompressed format that our Matlab/ImageJ could work with. The first 125 frames were truncated for the verification test. AR-DIC was run first with no adaptation and then with the adaptive reference scheme with iteratively obtained threshold of 3.5. Without the adaptive reference the small contractions near the relaxed state are obscured in the higher noise (**Fig. S12a**). With adaptive reference the small displacements are visible in the gradual slope after the relaxation. Even with potential compression artifacts, our AR-DIC method extracted useful information about the beating regions, beating area, and regions of strain (**Fig. S12**), indicating good agreement with their beating pattern analysis.

Then, tissue development over time was further quantified by processing the day 9 and day 10 videos from Rajasingh et al.[6] and comparing these results to day 8. These videos recorded the beating of the same region of tissue over the course of three days (culture days 8, 9, and 10). From day 8 to day 9, the increase in contraction displacement and contracting area is well quantified in the increased magnitude of the contraction volume (**Fig. S13a** left and center). Our methods could observe the original day 8 contraction center (**Fig. S13b**, O’ in red) integrating with a secondary region on day 9 (O’’). The result may indicate a relatively unsynchronized tissue with increased displacement magnitude, while the contraction initiation was shared between the original initiation site and the secondary region. The dyssynchrony was evident in the exaggerated relaxation phase in the day 9 contraction volume. A third contraction region had also appeared (O’’’) distal to O’ and O’’ on day 9. By day 10, no new contraction centers had developed. However, the existing contraction regions showed synchronized motion resulting in a uniform contraction volume compared to day 9. The improved tissue function was evident in the contraction volume magnitude which had almost doubled compared to day 8. These results from our AR-DIC assessment may provide additional insight into the tissue development process.

Comparing pixel sizes and reference frames

AR-DIC was tested with a vector spacing of 2 pixels, and examples of high resolution displacement and velocity plots are shown (**Fig. S14**). No significant increase in useful data outputs was obtained but with increased calculation time.

The 2D-FFT was applied to reference frames for verification of video sample quality for DIC. In 2D-FFT plots of reference images from videos of non-contracting control and contracting sample (**Fig. S15**), the features of comparable size and quality are evident confirming that reference images are comparable in quality. Displacements in CM contraction were also cross-checked manually in the Leica microscopy software by marking significant features and measuring the displacement changes in subsequent frames (not shown).

Supplementary Video 1

Video for beating CM sample.

Link is given at: https://unl.box.com/s/4il0vcdg7ird2z8ybazkzwn4iy64x0yg

Supplementary Video 2
Video for non-contracting CM control.
Link is given at: https://unl.box.com/s/ssb1cau9h2y1rimp56zwh6qh1id3pnl2

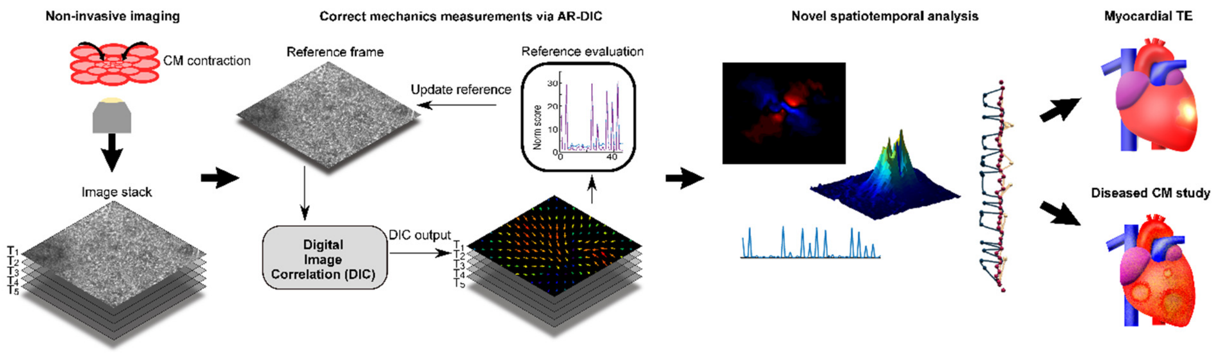

Fig. S1.
AR-DIC can be used to assess the functional development in myocardial tissue engineering (TE) and assist the functional assay of CM disease model with novel spatiotemporal characterizations and visualizations. Our analysis provides detailed quantification of parameters relevant to the functionality and maturity of engineered cardiac tissues such as the contracting area, relative tissue synchronization, discovery of contraction roots, interdependence of contracting regions, contraction kinematics, and tissue strain. The method may be used to gauge contractile tissue development in a non-invasive way, and provide detailed quantification and visualization to researchers who may apply the data to optimize TE protocols, test CM-drug interactions, or conduct mechanism studies.

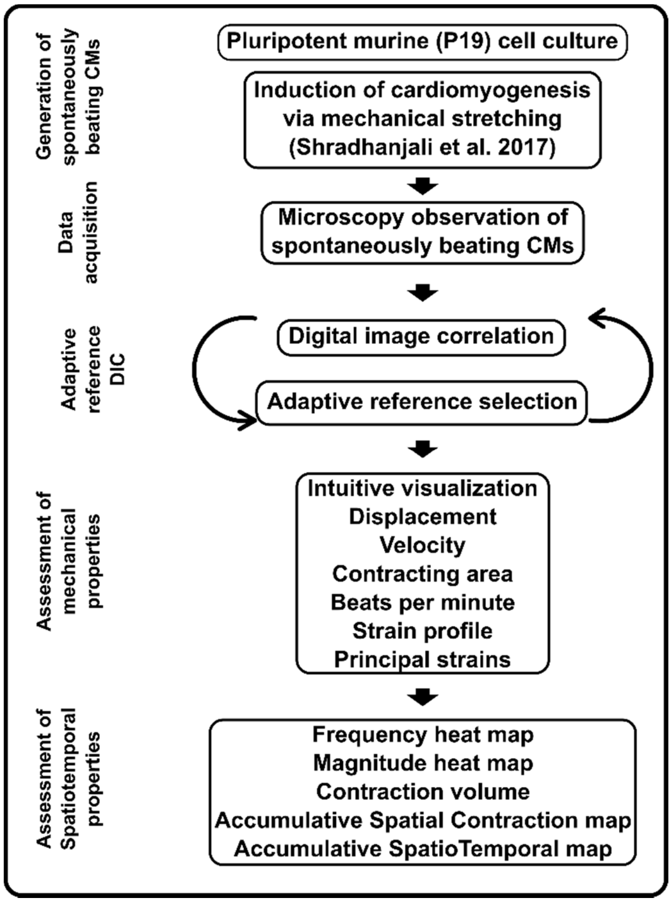

Fig. S2.
Schematic of the methodology utilizing video microscopy, digital image correlation with a novel adaptive reference approach, and custom built Matlab programming to obtain a range of kinematics properties and multiple new spatial and temporal parameters of CM contraction.

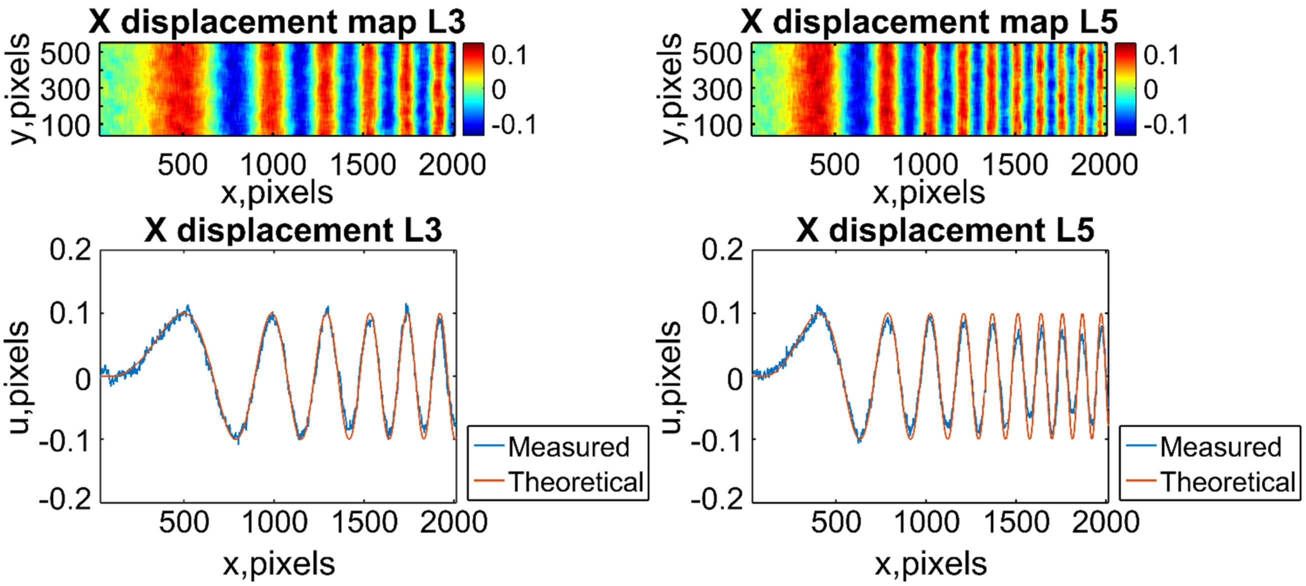

Fig. S3.

Examples of our AR-DIC applied to L3 and L5 from ‘Sample 14’ DIC Challenge from the Society for Experimental Mechanics (sem.org/dicchallenge/).

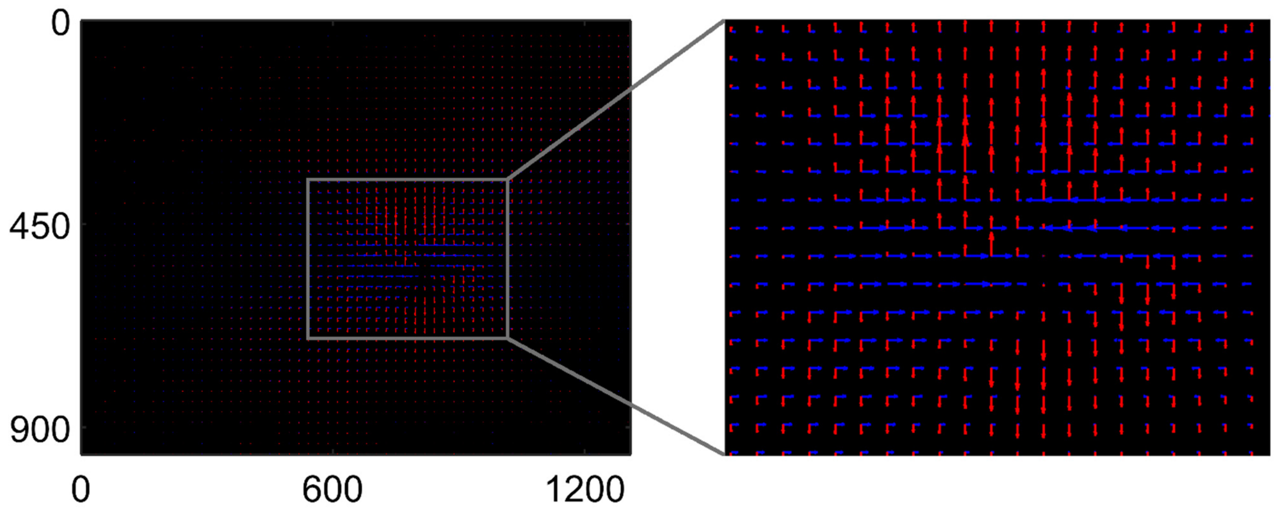

Fig. S4.
From **Fig. 2A**, decomposed *x* and *y* components of the displacement are shown. Red and blue arrows mark vertical and horizontal displacements, respectively. Frame units are in µm.

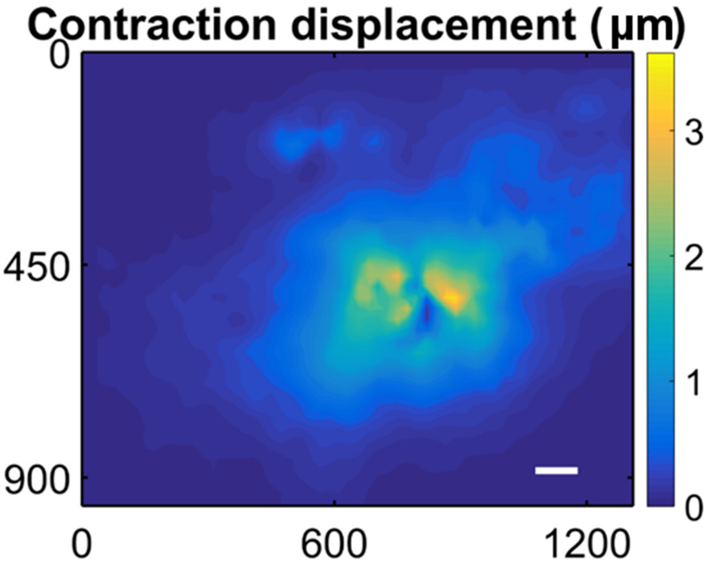

Fig. S5.
The displacement at each time frame throughout the video was obtained. The plot shown is the displacement at the frame showing the maximum beating area. See **Fig. 2A** for the displacement at the frame showing the maximum displacement, which is different from the image shown here. Frame units are in µm. Scale bar is 100 µm.

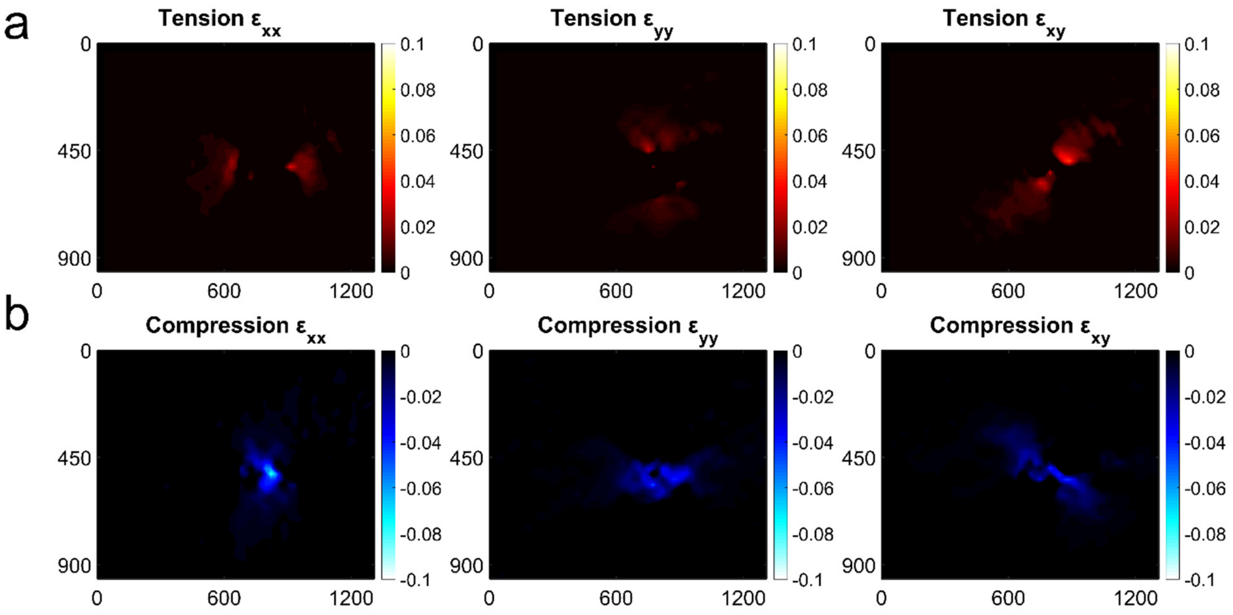

Fig. S6.
Tension (a) and compression (b) components of the strain at the maximum displacement frame for the contracting sample. Frame units are in µm.

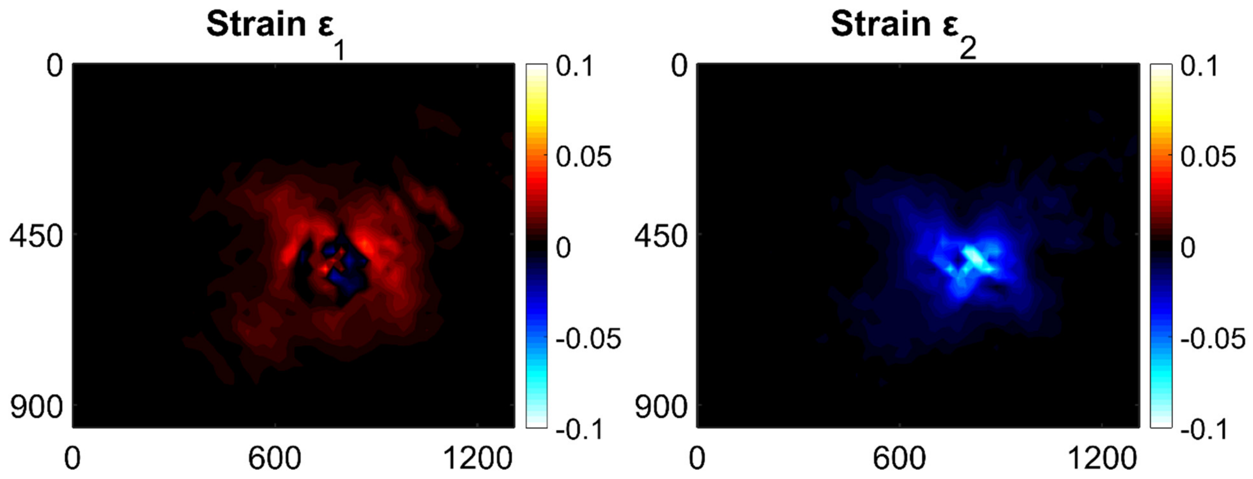

Fig. S7.

Principal strain fields plotted to show variation in maximum strains. Complementary to **Fig. 2I-K**, these plots reveal the maximum strain magnitudes, but the spatial interpretation of principal strains is limited since each sampled location requires a principal angle rotation. The contraction origin appears to have compression only and lacks tension. Frame units are in μm.

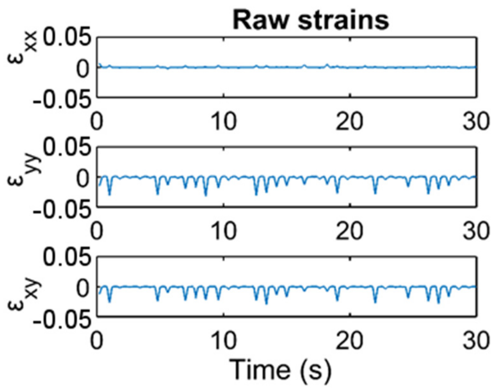

Fig. S8.

Raw strain values at the maximum principal strain location were plotted over time. From these raw strains, the principal strains were obtained as in **Fig. 2L**.

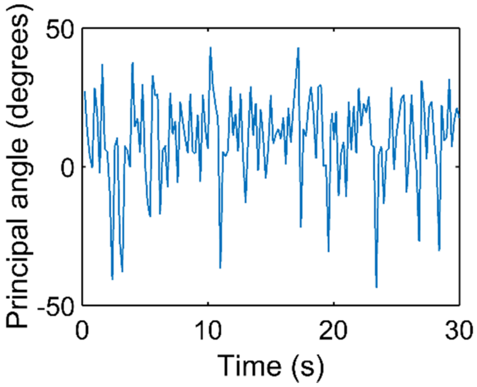

Fig. S9.

Principal angles for contracting samples, corresponding to the principal strains in **Fig. 2L**. The principal angle is the angle the local strain needs to be rotated to obtain the maximum principal strains.

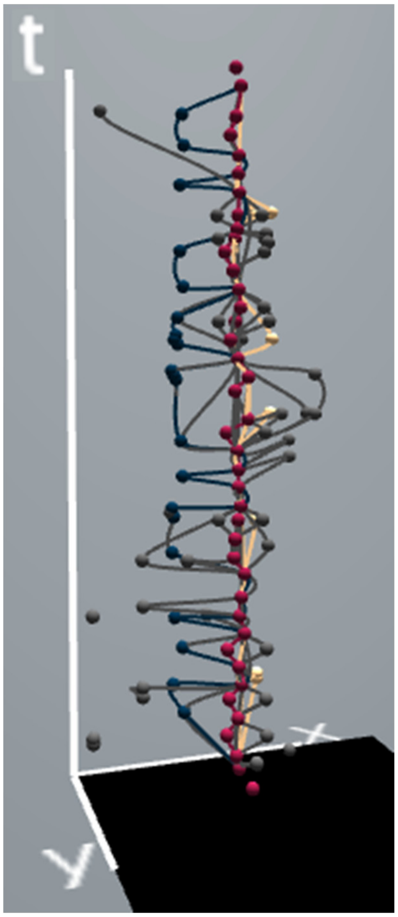

Fig. S10.
ASTC map for all identified contracting areas is plotted to illustrate the beating propagation and synchronization among the nodes.

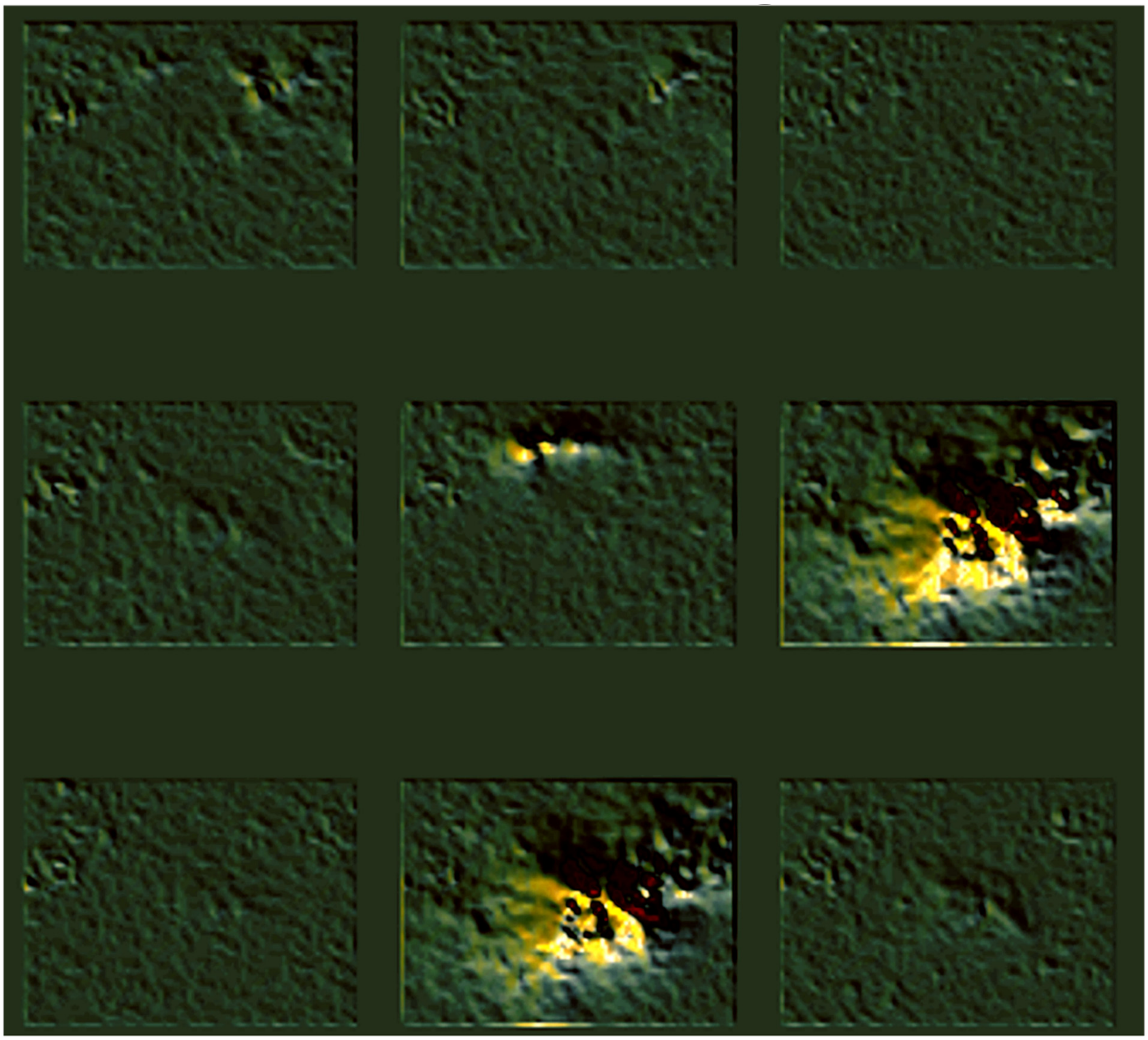

Fig. S11.

Tiled synthetic topography frames from the contracting sample. Elevated regions representing higher magnitudes of contraction are plotted as synthetic mountains from a bird's-eye-view. Individual video frames are tiled left to right, top to bottom to view the progression of contraction.

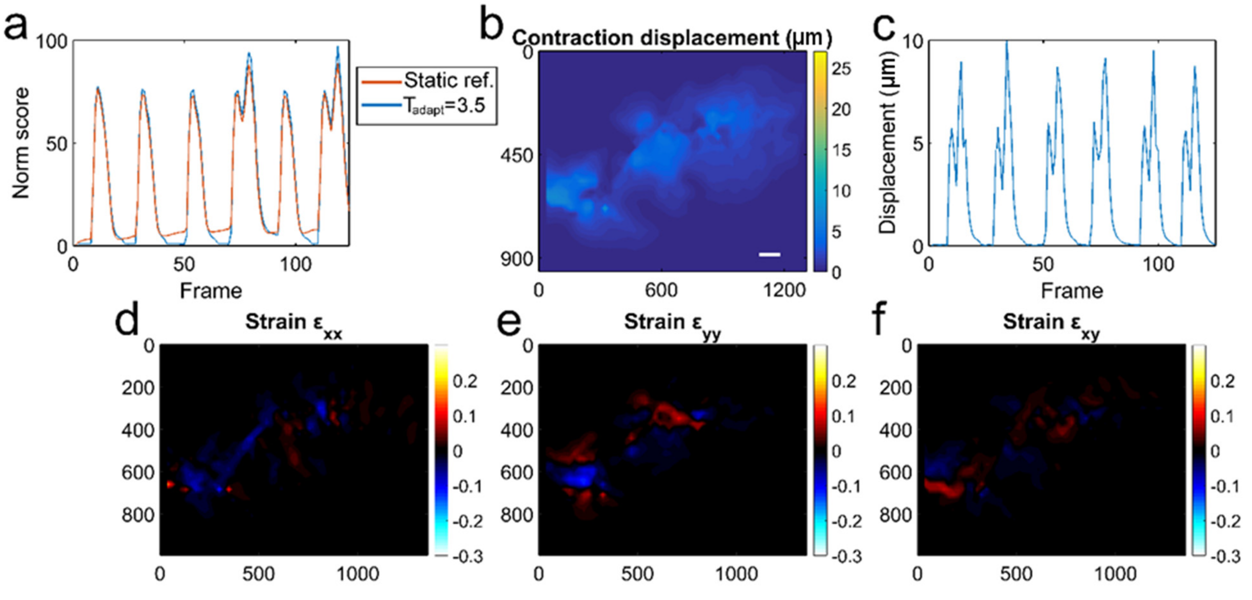

Fig. S12.

Our AR-DIC method was applied to the first 125 frames of "S2 Movie: Day 8 high frame rate video microscopy image of iPSC-CMs…" from Rajasingh et al.[6] (a) AR-DIC method prevented noise buildup in measurement. (b) AR-DIC could reveal the outline of the contracting area with displacement shown at the maximum contraction area frame. (c) AR-DIC was used to plot the displacement near the contraction origin. A comparison with Fig. 7D2 from Rajasingh et al.[6] shows good agreement. However, Rajasingh et al. used a static frame for DIC reference which limited the accurate determination of displacement magnitudes. (d-f) AR-DIC allowed the strain calculation, which was not available in the authors' original work[6]. At the frame of maximum contraction area, similar to our study, the main contraction origin is primarily in compression as seen in the bottom left portion of the ε_{xx} and ε_{yy} plots. Frame units are in µm. Scale bar is 100 µm.

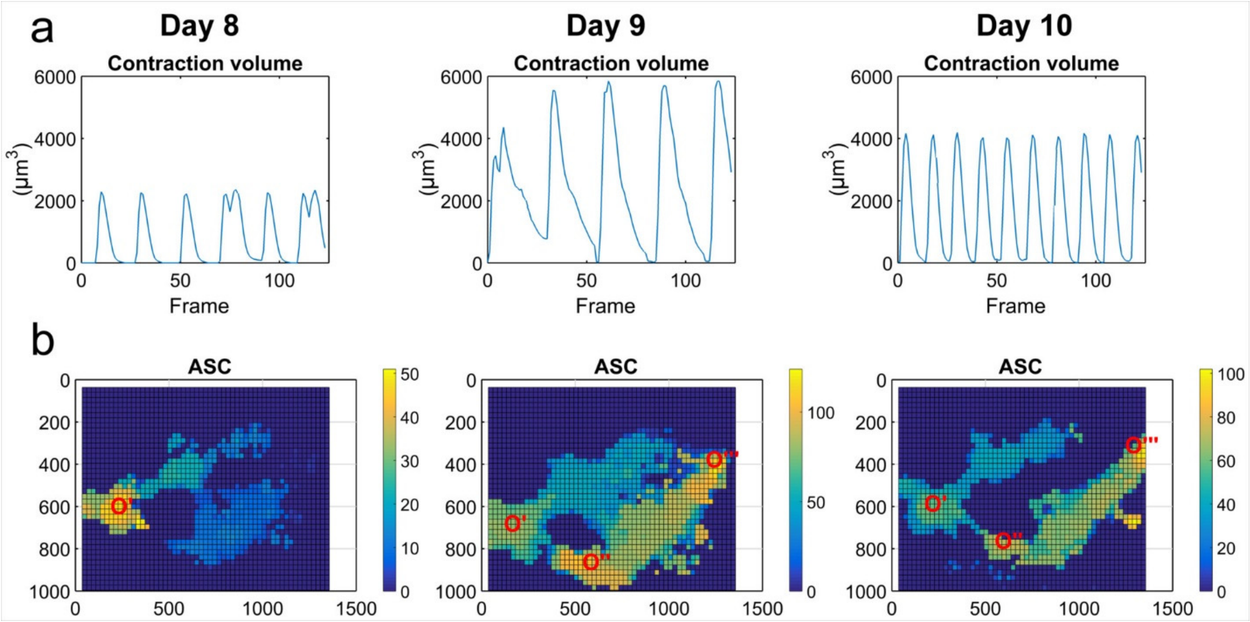

Fig. S13.

Tissue development over time was quantified by processing 125 frames from videos for culture days 8, 9, and 10 from Rajasingh et al[6]. Day 8: left, Day 9: center, Day 10: right. (a) Contraction volume increased from day 8 to day 9 with dyssynchrony evident in the relaxation phase of day 9. By day 10, the tissue regions were synchronized resulting in a uniform contraction volume. Contraction volume magnitude almost doubled from day 8 to day 10, which highlights the increased tissue function. (b) Our AR-DIC method was used to obtain the ASC map with color bar corresponding to the depth of the tree data. The main contraction center (O' in red color) is identified in the top-down view of the ASC map on day 8. By day 9, secondary (O'') and tertiary (O''') initiation sites had developed but were unsynchronized as evident in the contraction volume. On day 10, the three contraction sites had synchronized and the large tissue contractions were located primarily between the contraction centers. For clarity, only displacements above 3 μm were included in the ASC maps. Frame units are in μm.

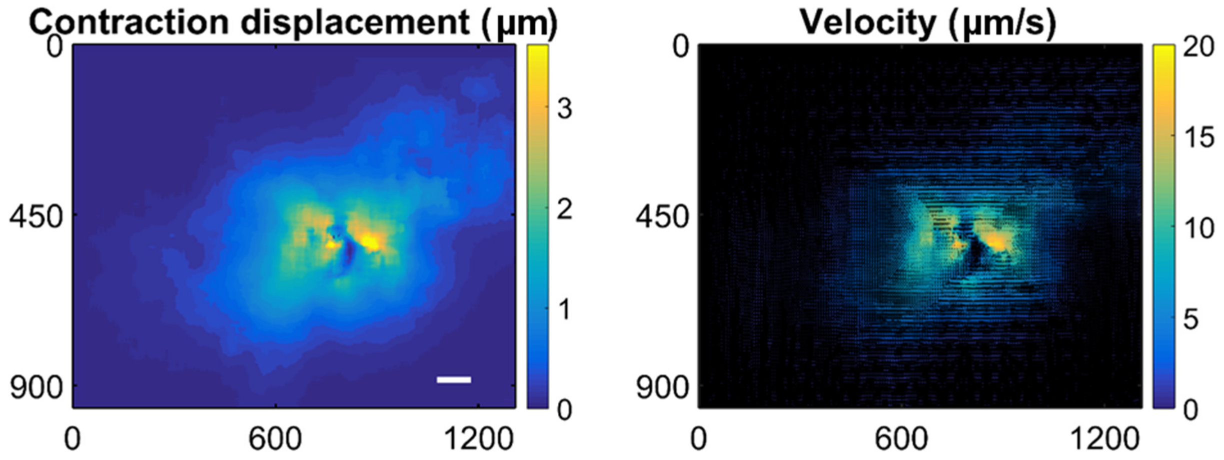

Fig. S14.

AR-DIC was tested with a vector spacing of 2 pixels. Examples of high resolution displacement and velocity plots at the maximum displacement frame are shown, with no significant increase in useful data outputs but with increased calculation time. Frame units are in μm. Scale bar is 100 μm.

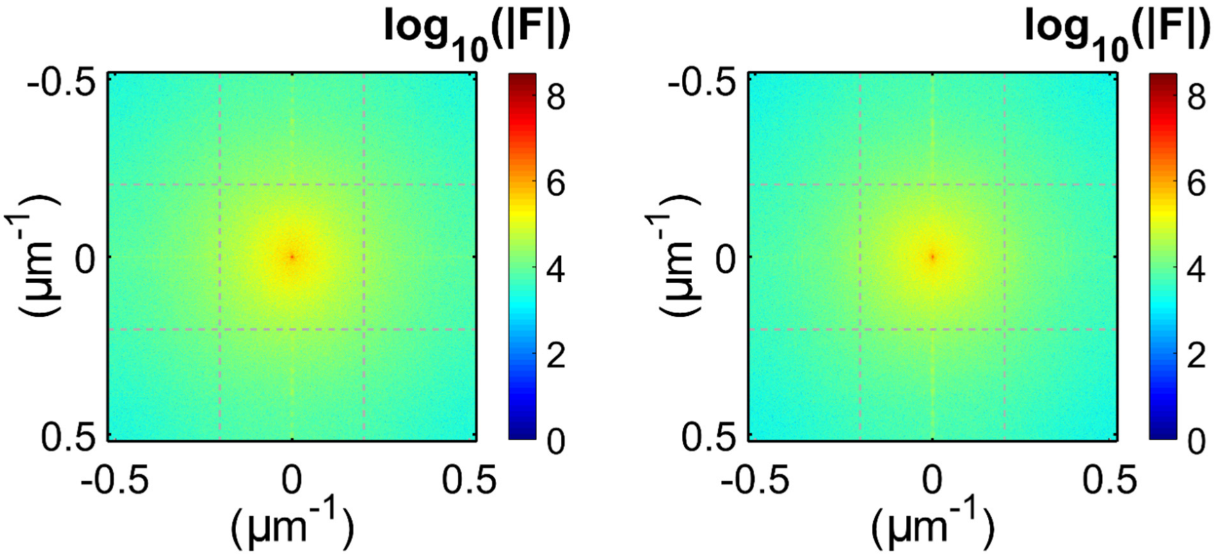

Fig. S15.

Power spectrum analysis of non-contracting sample (left) and contracting (right) video images are shown, which confirm that the adaptively selected reference images have comparable feature size and quality. A 2D-FFT was applied to reference frames for verification of video sample quality for DIC. For the reference frame, the Fourier transform is denoted as F and the power spectrum of its 2D FFT as |F| (shown at a logarithmic scale). Due to FFT transform, the axis units describe the spatial frequency (μm^{-1}). The smallest significant feature size was approximately 5 µm. Gray dashed lines are superimposed to mark the spatial frequency of 0.20 μm^{-1} corresponding to a feature size of 5 µm.
